# 

**Published:** 1883-02-20

**Authors:** 


### EDITORIALS AND MISCELLANEOUS.

### *EDITORIAL NOTICES.*

THE INDEX for the volume of 1883 was bound with our January No., but can be removed without mutilating the reading matter of that issue.

THE NEW YORK MEDICAL JOURNAL AND OBSTETRIC REVIEW, so long and favorably known as a monthly, is now being issued as a weekly journal. We regard it as among the very best in our large list of exchanges.

DR. J. THAD. JOHNSON, Professor of Surgery in the Southern Medica College, has, we regret to state, been extremely ill for some weeks past, and serious fears are entertained as to his recovery. His Chair in the College has been filled by Prof. Geo. G. Crawford.

A A. MELLIER.—See advertisement of this splendid establishment of St. Louis, Missouri, in this Journal. The Medical student and the practitioner should examine the Elliot saddle-bag, sold by this house. It is the best in the market.

CREDIT TO WHOM CREDIT IS DUE.—In our January number a clinical report from the "Service of F. L. T. Weir, M.D., Clinical Professor of Dermatology, should have been credited to The Medical and Surgical Reporter, Philadelphia.

BEAUTIFUL SAMPLES.—We are in receipt of samples of sugar coated pills of quinine, etc., from the House of Wm. R Warner & Co., Philadelphia. We have tested Warner's preparations and believe them to contain pure and select drugs, full measure, and to be carefully prepared. We feel safe in recommending them to the profession.

THE COMMENCEMENT EXERCISES of The Southern Medical College are appointed for the evening of Tuesday, February 27, 1883, at the Opera House in Atlanta. The degrees will be conferred by Prof. Powell, President of the Board of Trustees. The annual address will be delivered by Rev. Mr. McDowell. of Atlanta. The valedictorian of the class is Mr. A. S. Dyer, of Georgia.

MEDICAL ASSOCIATION OF GEORGIA.—The Medical Association of Georgia will meet the present year on the third Wednesday of April, at Athens, Georgia.

It is anticipated that there will be a large attendance, and that many matters of interest and importance will come up for consideration. Athens is a pleasant place, and renowned for the refinement, intelligence and hospitality of her citizens, and as this is the first time the Association has been appointed for that place we doubt not that the members will be warmly welcomed both by the citizens and the profession.

PARKE, DAVIS & Co.—See the new advertisement of this great drug house on 4th cover page. The variety, beauty and extent of their preparations are unsurpassed, and what is better, their preparations have an established reputation for purity and efficiency.

This house has done more in the introduction of new therapeutic agents than, perhaps, all others combined, and the result of their labors in this department have added largely to the armamentarium of the practitioner.

McKESSON & ROBBINS.—See the advertisement of this staunch and reliable house in this Journal. Their preparations are most excellent, and they have an established and wide-spread reputation throughout the entire country. Their Pills and Granules; their Saccharated Pepsin, Cod-Liver Oil, etc., are neatly and elegantly prepared, from pure drugs, and most admirable and convenient for the practitioner.

SHARPE & DOHME.—These reliable and enterprising manufacturing chemists of Baltimore have established a widespread and deserved popularity throughout the country. Their preparations of every kind are excellent, and are prepared with great care, neatness and precision.

SHARPE & DOHME'S POCKET DRUG CASE is among the very best in the market, as we have been kindly furnished with a sample containing twenty-four one-drachm vials, all filled with granules and sugar-coated pills most beautiful to look upon, and so well assorted and graded in the dose as to charm the eyes and delight the heart of the practitioner.

See advertisement of this house on second page of our Journal.

### *PAMPHLETS RECEIVED.*

THE PHYSIOLOGY OF ALCOHOLICS—An address by Wm. B. Carpenter, M.D., L.L. D., F. R. S., author of Principles of Human Physiology, Mental Physiology, etc., etc. New York: National Temperance Society and Publishing House, 58 Read street.

SCROFULA AND ITS DISEASES—An introduction to the general pathology of Scrofula, with an account of the histology, diagnosis and treatment of its glandular affections, by Fredrick Treves, T. R. C. S., Esq., Assistant Surgeon at the London Hospital, and late Professor of Pathology at the Royal College of Surgeons.

WHAT SHALL WE DO FOR THE DRUNKARD?—A rational view of the use of Brain Stimulants, by Orpheus Everts, M.D., Superintendent of the Cincinnati Sanitarium; late Superintendent of the Indiana Hospital for the Insane, etc. Cincinnati: Robert Clarke & Co., 1883.

SOME AFFECTIONS OF THE ORGANS OF RESPIRATION—In which the syrup of hypophosphites (Fellows') is beneficial. For the Medical profession, part II. London: 1883, Jas. I. Fellows, 48 Vesey street, New York.

### RECEIPTED.

To JANUARY, 1883.—Drs. W E Courson. J T Cleveland, T J Brasher, J W. Plummer, C D Tatman, J B Marshall, J L Hudson, J F Price, J W Ethridge, E Y Flemming, F M Fitzhugh, L B Bouchelle, W R Chambers, L D Johnson, to Jan. '82.

To JANUARY, 1884.—Drs. C H Jones, R F Mathews, J J Cunningham, to April '83; A J Kolb, C L Griffith, to July, '83; J E Frippe, B F Herrick, T L Turk, P C Tircuit, A H Redding, S J Welsh, L S Brownlee, G W Luster, J B Vandergriff, J T McDowell, J R Johnson, J O Landers, D B Hamilton, T S Parrham, J F Pou, J N Gilmore, to July,—; L H Hill, A Atkinson, J W Hoff, J H Hysel, J R Wilson, Lindsay & Lindsay, C J Burroughs, Louis Hadden. B L Clarke, R R Lyons, Hays Bros.

Dr. J C Terrell, to July '82; Dr. R D Lucius, 1882. Dr. L W Coleman, 1883.

